# Myc and Max Genome-Wide Binding Sites Analysis Links the Myc Regulatory Network with the Polycomb and the Core Pluripotency Networks in Mouse Embryonic Stem Cells

**DOI:** 10.1371/journal.pone.0088933

**Published:** 2014-02-21

**Authors:** Anna Krepelova, Francesco Neri, Mara Maldotti, Stefania Rapelli, Salvatore Oliviero

**Affiliations:** 1 Human Genetics Foundation (HuGeF), Torino, Italy; 2 Dipartimento di Biotecnologie Chimica e Farmacia Università degli Studi di Siena, Siena, Italy; 3 Dipartimento di Scienze Della Vita e Biologia dei sistemi, Torino, Italy; St. Georges University of London, United Kingdom

## Abstract

Myc is a master transcription factor that has been demonstrated to be required for embryonic stem cell (ESC) pluripotency, self-renewal, and inhibition of differentiation. Although recent works have identified several Myc-targets in ESCs, the list of Myc binding sites is largely incomplete due to the low sensitivity and specificity of the antibodies available. To systematically identify Myc binding sites in mouse ESCs, we used a stringent streptavidin-based genome-wide chromatin immunoprecipitation (ChIP-Seq) approach with biotin-tagged Myc (Bio-Myc) as well as a ChIP-Seq of the Myc binding partner Max. This analysis identified 4325 Myc binding sites, of which 2885 were newly identified. The identified sites overlap with more than 85% of the Max binding sites and are enriched for H3K4me3-positive promoters and active enhancers. Remarkably, this analysis unveils that Myc/Max regulates chromatin modifiers and transcriptional regulators involved in stem cell self-renewal linking the Myc-centered network with the Polycomb and the Core networks. These results provide insights into the contribution of Myc and Max in maintaining stem cell self-renewal and keeping these cells in an undifferentiated state.

## Introduction

The nuclear factor c-Myc (Myc) is a basic helix-loop-helix leucine zipper (bHLHZ) transcription factor that binds the consensus DNA sequence known as the E-Box (CACGTG) when dimerized with Max [1]. This dimer regulates the transcriptional activation of target genes. Myc is a master regulatory transcription factor that has been estimated to bind to over 15% of all promoters in different cell types, modulating the expression of a large number of its target genes [2]. Myc is considered a global gene regulator that acts by recruiting enzymes to the chromatin that induce covalent modifications in histone tails [3], [4]. In response to environmental stimuli, Myc modulates a large number of cellular processes, such as proliferation, growth, differentiation, metabolism, and even apoptosis [3]. Myc also plays a role in ES cell pluripotency [5]–[8]. Chromatin immunoprecipitation coupled with massive parallel sequencing (ChIP-Seq) is a powerful method for the identification of binding sites of chromatin-associated proteins, and several experiments have been performed to identify Myc binding sites [6], [9]–[13]. However, ChIP experiments are limited by the specificity of the antibody used and the degree of enrichment achieved in the immunoprecipitation step. The list of identified genes to which Myc binds is largely incomplete, mainly because genome-wide analyses of Myc binding sites are hampered by the quality of the available antibodies. One way to circumvent this problem is the expression of epitope-tagged proteins.

To generate a more comprehensive map of Myc binding within the genome in mouse embryonic stem cells (ESCs), we compared the chromatin immunoprecipitation (ChIP) efficiency of four affinity tags. We generated ESC clones expressing Myc tagged at its N-terminus with either a Biotag, a FLAG-HA, or a V5 epitope and compared the efficiency and selectivity of each in ChIP experiments under different conditions. A genome-wide analysis was performed to compare the results of Bio-Myc ChIP-Seq with previously published ChIP-Seq data obtained with an antibody recognizing endogenous Myc [9]. We identified a large number of Myc binding sites that were previously undetected. Because Myc must form a dimer with Max to bind to an E-Box element, we also performed ChIP-Seq with Max and found that Myc with Max share over 85% of their genomic binding sites. The validation of a group of newly identified genes showed that these genes are actually bound and regulated by Myc in ESCs.

## Materials and Methods

### Cell culture conditions

Mouse embryonic stem cells (ESCs) cells were cultured in DMEM high glucose medium (Invitrogen) supplemented with 15% FBS (Millipore), 0.1 mM nonessential amino acids (Invitrogen), 1 mM sodium pyruvate (Invitrogen), 0.1 mM 2-mercaptoethanol, 1500 U of LIF/ml (Millipore), 25 U of penicillin/ml, and 25 µg of streptomycin/ml.

### DNA constructs

The cDNA of Myc was cloned into the pEF6/V5-His vector. Myc was N-terminally tagged by introducing into the *Kpn*I and *Spe*I sites of pEF6 vector different tag peptides: Biotag, V5 tag or FLAG-HA tandem epitopes. Short hairpin RNA (shRNA) constructs were purchased by Open Biosystems: Myc shRNA 1 (TRCN0000086913), and NMyc shRNA 1 (TRCN0000020694).

### Generation of mouse BirA-ES cell lines stably expressing tagged Myc

To obtain tagged BirA-Myc stable clones, BirA-ESCs were transfected with linearized Myc constructs using Lipofectamine 2000 Transfection Reagent (Invitrogen) according to the manufacturer's protocol. Transfected cells were cultured for ten days in growth medium with Blasticidin and drug-resistant clones were selected for Myc expression.

### Nuclear protein extraction and immunoprecipitation

Cells were harvested in 1× PBS and resuspended in an isotonic buffer (20 mM HEPES pH 7.5, 100 mM NaCl, 250 mM Sucrose, 5 mM MgCl_2_, 5 µM ZnCl_2_). Then, the cells were resuspended in an isotonic buffer supplemented with 1% NP40 to isolate the nuclei. The isolated nuclei were resuspended in digestion buffer (50 mM Tris-HCl pH 8.0, 100 mM NaCl, 250 mM Sucrose, 0.5 mM MgCl_2_, 5 mM CaCl_2_, 5 µM ZnCl_2_) and treated with micrococcal nuclease at 30°C for 10 min. The nuclear proteins were incubated with 3 µg of the specific antibody overnight at 4°C. The immunocomplexes were incubated with protein G-conjugated magnetic beads (DYNAL, Invitrogen) for 2 h at 4°C. To immunoprecipitate tagged Myc, the specific anti-tag affinity beads were used. The samples were washed four times with digestion buffer supplemented with 0.1% NP-40 at RT. The proteins were eluted by incubation with 0.4 M NaCl TE buffer for 30 min and analyzed by western blotting.

### Chromatin Immunoprecipitation (ChIP) assay

Approximately 2×10^7^ cells were cross-linked by addition of formaldehyde to 1% for 10 min at RT, quenched with 0.125 M glycine for 5 min at RT, and then washed twice in 1× PBS. The cells were resuspended in Isotonic buffer supplemented with 1% NP-40 to isolate nuclei. The isolated nuclei were then resuspended in ChIP Buffer (20 mM Tris-HCl pH 8.0, 150 mM NaCl, 2 mM EDTA and protease inhibitors) supplemented with different concentration of detergents: 0% SDS, 1% Triton X-100; 0.15% SDS, 1% Triton X-100; 0.5% SDS, 0.5% Triton X-100; and 1% SDS, 0% Triton X-100.

Extracts were sonicated using the Bioruptor® Twin (Diagenode) for 2 runs of 10 cycles [30 sec “ON”, 30 sec “OFF”] at high power setting. Cell lysate was centrifuged at 12,000 g for 10 min at 4°C. The supernatant performed in the conditions 3 and 4 was diluted with ChIP Dilution Buffer (20 mM Tris-HCl pH 8.0, 150 mM NaCl, 2 mM EDTA, 1% Triton) before immunoprecipitation step.

Bio-ChIP. Streptavidin beads (Dynabeads® MyOneTM Streptavidin T1) were saturated with PBS/1% BSA at RT for 1 hour, and then incubated with sample at 4°C for 4 hours on a rotator. Immunoprecipitated complexes were successively washed with Washing Buffer I (2% SDS), Washing Buffer II (50 mM HEPES pH 7.5, 500 mM NaCl, 0.1% Deoxycholate, 1% Triton X-100, 1 mM EDTA), Washing Buffer III (10 mM Tris-Cl pH 8.1, 250 mM LiCl, 0.5% NP-40, 0.5% Deoxycholate, 1 mM EDTA,) and TE buffer (10 mM Tris-HCl pH 7.5, 1 mM EDTA). All washes were performed at RT for 8 min on a rotator. SDS Elution Buffer (50 mM Tris-HCl pH 8.0, 10 mM EDTA, 1% SDS) was added and incubated at 65°C overnight to reverse crosslink protein-DNA complexes. After decrosslinking, DNA was purified using QIAQuick PCR Purification Kit (Qiagen) according to the manufacturer's instructions.

V5-ChIP. Anti-V5 tag-magnetic beads (MBL International) were saturated with PBS/1% BSA at RT for 1 hour, and then incubated with sample at 4°C for 16 hour on a rotator. Immunoprecipitate complexes were washed as described previously [8], eluted with SDS Elution Buffer or with V5 peptide (Sigma), and then decrosslinked and purified as described above.

FLAG ChIP was performed as V5-ChIP using anti-FLAG M2 magnetic beads (Sigma) for immunoprecipitation and 3×FLAG peptide (Sigma) or SDS Elution Buffer for elution.

For tandem FLAG-HA ChIP, we first immunoprecipitated the sample by incubating with anti-FLAG M2 magnetic beads (Sigma) at 4°C for 16 hours on a rotator, then washed and eluted the immunoprecipitated complexes with 3×FLAG peptide (Sigma) and reincubated the eluate with EZview Red Anti-HA Affinity Gel (Sigma) at 4°C for 4 hr. After five washes, SDS Elution Buffer or HA peptide (Sigma) was added, and DNA was decrosslinked and purified.

For reference sample, BirA-ES cells without tagged protein were used.

ChIP experiments against endogenous Max, Myc, NMyc and V5-tag (using antibody) were performed as previously described [8]. Briefly, extracts were incubated with specific antibodies overnight and the immunocomplexes were captured using saturated protein G magnetic beads in gentle rotation for 2 h at 4°C. After 5 washes, the complexes were eluted in SDS Elution Buffer and DNA was decrosslinked and purified. IgG was used as a negative control.

### RT-qPCR analysis

For the ChIP experiments, immunoprecipitated DNA was analyzed by quantitative real-time PCR using the SYBR GreenER kit (Invitrogen). The oligonucleotide sequences are indicated in Table S1.

RNA was extracted and quantified as previously described [14]. The RNA was analyzed by quantitative real-time PCR using the Superscript III Platinum One-step qRT-PCR System (Invitrogen). The oligonucleotide sequences are indicated in Table S2.

### Genome-wide bioinformatics analysis

A library was constructed from the eluted DNA using the ChIP-Seq Sample Prep Kit (Illumina) and sequenced on the Illumina HiScanSQ Platform. The reads from sequencing were mapped to the mouse genome (mm9 assembly) using Bowtie version 0.12.7, reporting only unique hits with up to two mismatches. The redundant reads were collapsed, and peak calling was performed using MACS version 1.4.1. For comparative analysis, we downloaded GEO data for ESC histone modifications (GSE12241 and GSE11172) and transcription factors (GSE11431, GSE24843, GSE26833). RNA-Seq data from ESCs was downloaded from the ENCODE project database [15]. The LCP-ICP-HCP promoters were defined as in [16], H3K4me3-only and bivalent promoters were defined as in [17], and active or poised enhancers were defined as in [18], [19]. The ChIP-Seq peak calling was performed with Macs Software [20] with normalization for mock or IgG ChIP at a fixed p-value = 1E-8. Heatmaps and comparative analysis were performed using custom Perl scripts. Motif discovery was performed using HOMER [21]. The ROC curves were generated by calculating the sensitivity and specificity for the called peaks at different p-values. For cumulative probability, we selected two groups of gene promoters to be modeled as background and foreground. For the Max target promoters, we defined the background as the promoters of the bottom 50% of the expressed genes that were not bound by Max, and the foreground was defined as the promoters of the top 50% of the expressed genes that were bound by Max. For the H3K4me3 target promoters, we defined the background as the promoters of the bottom 50% of the expressed H3K4me3-negative genes, and the foreground was defined as the promoters of the top 50% of the expressed H3K4me3-positive genes. We then calculated the cumulative probability of each ChIP-Seq raw data signal for both groups. Gene ontology analyses were performed using Panther Classification System [22], [23].

## Results and Discussion

### Comparison of different affinity tags for use in chromatin immunoprecipitation

Myc has been estimated to bind and regulate approximately 15% of all mammalian genes. However, due to the poor specificity and high background detected with Myc-specific antibodies, only a fraction of these sites have been previously identified. To obtain a comprehensive list of Myc binding sites in ESCs, we employed a tagging approach.

**Figure 1:**
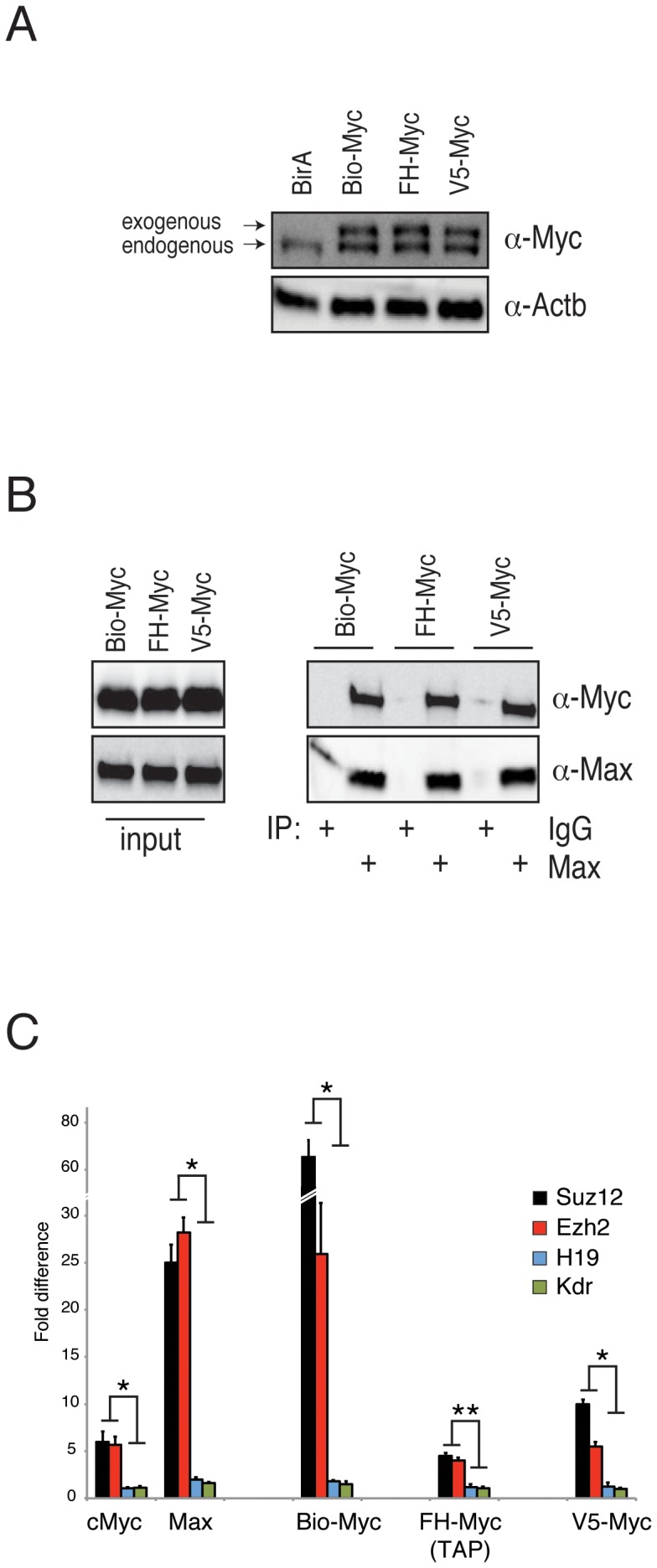
Characterization and functional validation of stable clones expressing tagged Myc. (A) Western blot analysis of the exogenous tagged Myc expression in the indicated stable clones. Actin (Actb) was used as a loading control. (B) Immunoprecipitation analysis of endogenous Max protein in nuclear extracts obtained from the indicated clones demonstrated that Max is able to co-immunoprecipitate with exogenous Myc in each clone analyzed. Purified rabbit IgG was used as a negative control. (C) RT-PCR analysis of ChIP assays of the indicated endogenous and tagged proteins. Suz12 and Ezh2 were used as positive controls, and H19 and Kdr were used as negative controls. The results are shown as the fold difference.

We first compared the efficiency of four affinity tags. We cloned Myc under the control of a ubiquitous EF1α promoter (pEF6 vector) that remains unmethylated in ES cells. We tagged Myc at its N-terminus with Biotag, FLAG/HA, or a V5 epitope. The FLAG and HA epitopes were cloned in tandem to perform a double consecutive ChIP for better specificity. We generated stable clones expressing each of the tagged Myc construct in ESCs. The clones maintained the typical ESC morphology and expressed the tagged Myc at a level comparable to the endogenous protein (Figure 1A). To functionally validate the clones, we performed co-immunoprecipitation experiments to verify that each tagged Myc formed a complex with the endogenous Max (Figure 1B and Figure S1).

To compare the binding to the chromatin of the different tagged Myc proteins, we performed chromatin immunoprecipitation (ChIP) on the Suz12 and Ezh2 promoters, which were previously shown to be Myc targets [8]. As negative controls, we used the H19 and Kdr promoters because these genes were shown not to be bound by Myc in previous genome-wide studies [9], and their transcription is nearly undetectable in ESCs by RNA-Seq. We performed ChIP analyses under different experimental conditions to establish the best parameters for each tag. The ChIP samples were analyzed by RT-qPCR, and the results were evaluated as the fold difference between the ChIP from cells expressing tagged Myc and the parental mock cells.

The biotin-streptavidin affinity allows the use of high SDS concentration in ChIP experiments. We performed ChIP analysis of the Bio-Myc clone using three different concentrations of SDS (0.15%, 0.5%, and 1%) in lysis buffer (Figure 1A). We observed good fold enrichment at the Suz12 and Ezh2 promoter regions with respect to the negative control regions under all SDS concentrations used. Very low background levels were observed in the Myc negative control regions as well as in Mock ChIP, most likely due to the high stringency of the washing buffer used in this protocol (Figure 1C and Figure S4A, B).

ChIP analysis of the FLAG could only be performed in the absence of SDS. Under these conditions, we were able to observe a 2- to 3-fold enrichment at the positive regions with respect to the negative control regions, while the ChIP with higher stringency lysis buffer (0.15% SDS) did not detect specific binding to the Suz12 or Ezh2 promoters (Figure S2B). Notably, we observed a high level of background in the Mock and negative controls in all FLAG-Myc ChIP experiments, suggesting that some nonspecific interactions occur in the FLAG immunoprecipitations of the chromatin (Figure S4A, B). To overcome this problem, we performed a double consecutive ChIP (TAP-ChIP). We first immunoprecipitated FLAG-HA-Myc with anti-FLAG M2 beads and eluted the immunoprecipitated complexes with a 3×FLAG peptide. We subsequently re-immunoprecipitated the eluate using anti-HA affinity gel. After the second immunoprecipitation, we eluted the DNA-protein complexes either with SDS buffer or with HA peptide. Under these conditions we were able to observed a more evident enrichment at the positive regions with respect to the negative control regions. We obtained up to fivefold enrichment at the positive regions in the ChIP experiments with a lower noise-to-signal level (Figure 1C and Figure S2C).

To immunoprecipitate V5-Myc we used anti-V5 magnetic beads and performed ChIP analyses under three different concentrations of SDS (0.15%, 0.5% and 1%). Lysis buffer containing 0.15% SDS gave better results. In addition, elution with the V5 peptide led to a higher enrichment in the positive control regions (Figure 1C and Figure S3A–C), while the ChIP of V5-Myc using the antibody against V5 conjugated to Protein G magnetic beads did not show enrichment (Figure S3B). The ChIP of V5-Myc showed a low level of background, especially when eluted with the V5 peptide, thus demonstrating its suitability for this type of application (Figure S4A–C).

In conclusion, ChIP performed by immunopurification using the V5 tag or double immunopurification using the FLAG-HA achieved levels of enrichment similar to those obtained with the anti-Myc antibody. However, the ChIP using the Biotag demonstrated much better performance than immunopurification with the specific antibody. Importantly, the Biotag showed the best signal-to-noise ratio, which is an important parameter to perform genome-wide ChIP assays.

### Genome-wide analysis of Bio-Myc identified a large number of Myc binding sites that overlap with Max

On the basis of the above results, we performed a genome-wide analysis using the Bio-Myc ChIP to identify Myc binding sites in ESCs. To bind DNA at E-Box elements, Myc must form a dimer with Max. We therefore also performed a ChIP-Seq analysis of Max and compared the genome-wide results for co-occupancy of Max with Myc based on the ChIP-Seq of Bio-Myc with the results obtained with Myc.

**Figure 2:**
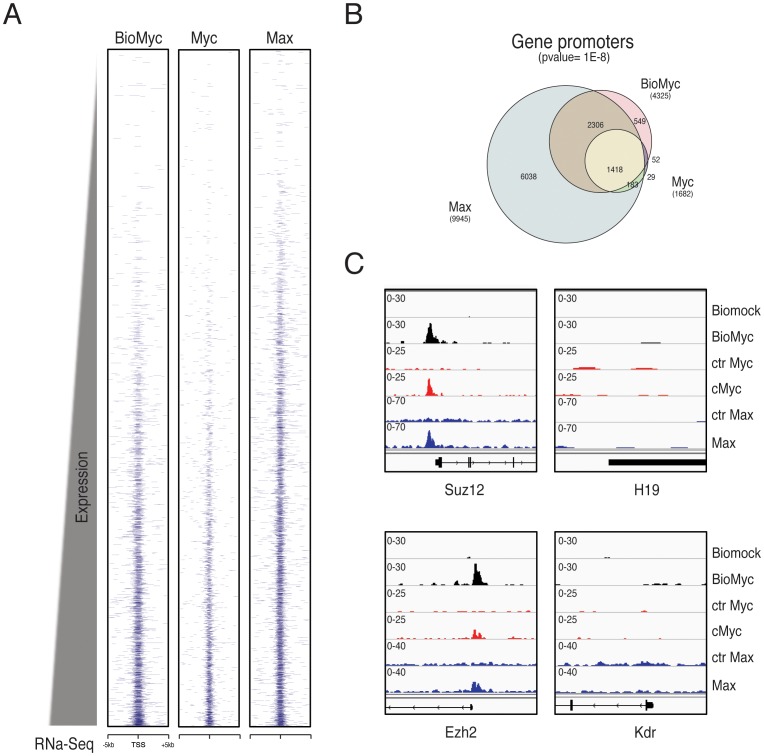
Comparison of Biotag-Myc versus anti-Myc antibody Chip-Seq. (A) Heatmap representation of ChIP-Seq binding for BioMyc, Myc, and Max around transcriptional start sites (TSSs) ±5 kb for all genes rank ordered from the lowest to the most highly expressed according to thee mRNA expression levels obtained from RNA-Seq analysis. (B) Venn diagram showing the exact number of promoters bound in the indicated ChIP-Seq analyses. Bio-Myc ChIP-Seq found many more genes bound on TSSs in comparison with the endogenous Myc ChIP-Seq (4325 TSS vs. 1682 TSSs, respectively), and Bio-Myc ChIP-Seq shows more overlapping genes with Max ChIP-Seq (2306 vs. 1418, respectively). (C) Genomic occupancy profiles of the indicated Chip-Seq show a well-defined peak of enrichment on the E-Box at the Suz12 promoter (−570 bp from TSS) and the Ezh2 TSS, while there is no binding at the promoters of the negative control genes (H19 and Kdr).

We generated heatmaps of the called peaks around the transcriptional start sites (TSS) ±5 kb of all genes rank-ordered by their mRNA expression levels obtained from the RNA-Seq analysis [15] (Figure 2A). As expected, the Bio-Myc peaks were enriched at the TSSs of highly expressed genes. Bio-Myc ChIP-Seq revealed a significantly larger number of binding sites on gene promoters with respect to the ChIP-Seq performed with antibodies recognizing endogenous Myc (Figure 2B). Importantly, the analysis of Bio-Myc binding sites showed a higher co-occupancy of Bio-Myc with Max if compared to Myc ChIP-Seq (Figure 2A, B). Examination of the binding profiles of Bio-Myc, Myc, and Max at the previously validated E-Boxes of the Suz12 promoter (−570 bp from TSS) and at the TSS of Ezh2 revealed a well-defined peak of enrichment for all three genome-wide analyses, while the analysis showed no binding at the promoters of the negative controls, the H19 and Kdr genes (Figure 2C).

**Figure 3:**
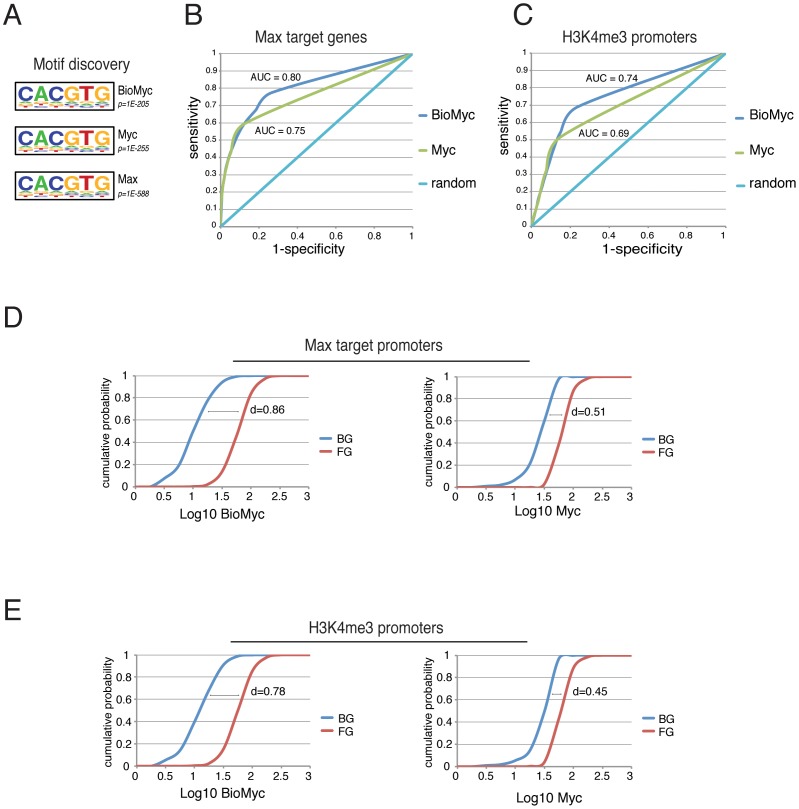
Biotag-Myc ChIP-Seq is more sensitive without loss of specificity. (A) Motif discovery analysis showed a significant enrichment for the E-Box sequence (CACGTG) in all the ChIP-Seq indicated. (B–C) The ROC curve highlights that Bio-Myc ChIP-Seq in comparison with Myc ChIP-Seq is more sensitive without a loss of specificity at Max or H3K4me3 target genes. The major area under the curve (AUC) for Bio-Myc indicates its better performance compared with Myc (AUC = 0.80 vs. 0.75 for Max target genes and AUC = 0.74 vs. 0.69 for H3K4me3 genes). (D–E) Bio-Myc (left panels) or endogenous Myc (right panels) ChIP-Seq reads cumulative frequency distributions are plotted for the background and foreground regions calculated for Max or H3K4me3 binding. A greater distance between the curves in Bio-Myc (d = 0.86 and d = 0.78) with respect to the endogenous Myc (d = 0.51 and d = 0.45) indicates a greater difference of signal intensity occurring between the background and bound regions.

It has been previously shown that overexpressed Myc could associate with low-affinity targets at an increased frequency and, at even higher levels, with other sequences [24] [25]. Motif discovery analysis of Bio-Myc, Max, and Myc ChIP-Seq analyses showed that all three ChIP-Seq datasets are enriched for a DNA sequence containing the perfect E-Box sequence CACGTG with high p-values: Bio-Myc 1E-205, Myc 1E-255, and Max 1E-588 (Figure 3A). Thus, although Bio-Myc ChIP-Seq identified many more binding sites, it maintained a similar enrichment of the motif sequence of the DNA-bound regions, suggesting that all sites identified by Bio-Myc ChIP–Seq are *bona fide* Myc binding sites and were not detected due to overexpression of the tagged protein.

We next divided all of the genes in two categories on the basis of their promoter binding by Max and calculated the sensitivity and specificity using Bio-Myc or the antibody anti-Myc at different p-values overlapping between the two categories. The ROC curve highlights that Bio-Myc ChIP-Seq is more sensible without loss of specificity, and it demonstrates the better performance of Bio-Myc ChIP-Seq in comparison with the ChIP-Seq performed with anti-Myc antibodies, as shown by the area under curve (AUC) (Figure 3B). We obtained similar results using promoters marked by H3K4me3 or by Max binding (Figure 3C). Furthermore, we generated two datasets of gene promoters. One used as a background (BG) of Myc binding sites comprised that is formed by 50% of the less genes with the lowest levels of expression among those expressed genes that were not bound by Max and, was used as the background (BG) for the Myc binding sites. The other, used as a foreground (FG)comprised composed of 50% of the genes with the highest levels of expression among the genes that were bound by Max and was used as the foreground (FG) most expressed genes bound by Max. The cumulative Bio-Myc or Myc signal distributions are plotted for the background or foreground regions. Interestingly, the BG curve of for Bio-Myc is more left- shifted in comparison with the BG curve of for Myc, indicating a minor density of reads in the background. Consequently, the distance (d) between the BG and FG curves of Bio-Myc is greater (Figure 3D). Again, using either H3K4me3 or Max, we found a similar distribution pattern (Figure 3E). Thus, Bio-Myc ChIP-Seq identifies a large number of Myc binding sites that could not be detected using the Myc antibody. This difference can be attributed to the high affinity of the biotin/streptavidin detection that can be performed at a very high stringency and to the very low background that is obtained using this detection system, which allows the identification of peaks that would not be distinguished from the background using the Myc- specific antibody.

### Biotag-Myc ChIP-Seq shows a high overlap with NMyc on promoters and enhancers

**Figure 4:**
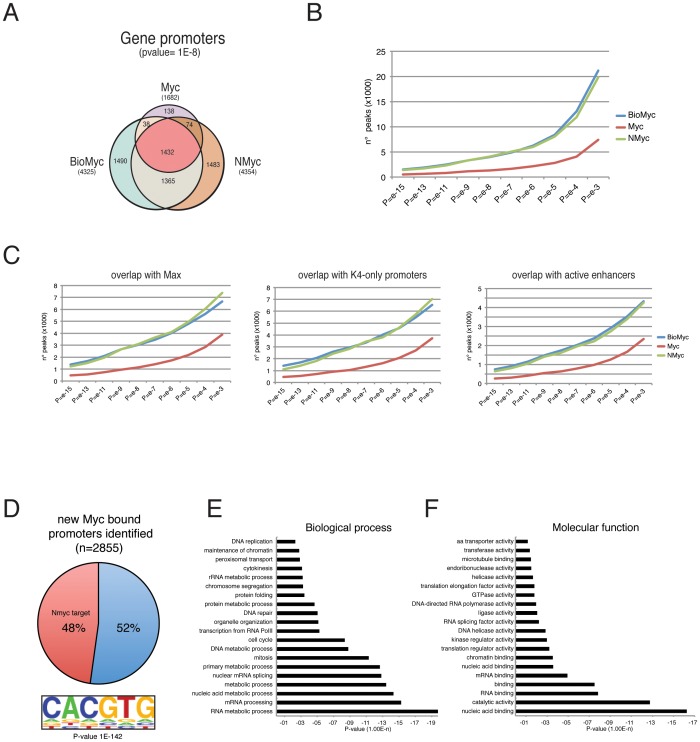
Biotag-Myc identifies a large number of Myc binding sites in ESCs. (A) Venn diagram showing the number of gene promoters bound in the indicated ChIP-Seq analyses and their overlap. (B) Bio-Myc ChIP-Seq was able to identify many more bound regions in comparison with Myc ChIP-Seq as shown by the number of peaks found at each p-value indicated. (C) The genomic colocalization of Bio-Myc peaks with Max, H3K4me3-only promoters, and active enhancers at different p-values. Bio-Myc ChIP-Seq always reveals a major number of peaks overlapping with the indicated genomic features and shows a similar distribution to the NMyc dataset. (D) About 50% of the newly identified Myc-bound promoters show overlap with NMyc targets and an enrichment in the motif sequence corresponding to the perfect Myc E-Box (p-value 1E-142). (E–F) Gene ontology analysis reveals that the newly identified Myc target genes are enriched for genes associated with the cell cycle and cell metabolism as well as genes with molecular functions involved in the metabolism of nucleic acid and chromatin structure.

As shown above, the high level of co-occupancy of Bio-Myc and Max strongly suggests that the biotin/streptavidin protocol identifies true Myc binding sites within the genome. Previous ChIP experiments showed that when coexpressed in the same cells, Myc and NMyc bind to the same promoters [8]. The analysis performed using the NMyc ChIP-Seq dataset [9] revealed that similarly to Bio-Myc, NMyc was able to identify a larger number of peaks when compared with the sites identified using the anti-Myc antibodies, most likely due to the major affinity and specificity of the antibody anti-NMyc. The overlap between the Bio-Myc and NMyc-bound regions was approximately 70%, demonstrating that the number of common binding sites between Myc and NMyc is larger than previously expected (Figure 4A, B). Our results indicate that the higher number of sites identified by NMyc compared with those identified using the anti-Myc antibody is not due to a different binding specificity of the two transcription factors, but to the higher specificity of the anti-NMyc antibody. These results suggest that the genome-wide approach may underestimate the number of total binding sites of a specific protein, especially in the cases when the antibody used has a low affinity and/or specificity. In these cases, the use of a tag represents a better approach to identify *bona fide* binding sites. In fact, while we were able to observe the Myc and NMyc binding to all these promoters by ChIP at each locus by RT-qPCR amplification of the fragment, only the use of Bio-Myc allowed us to identify these binding sites by genome-wide analysis.

The peaks identified by Bio-Myc and NMyc also show a higher overlap with open or active promoters marked by trimethylation of lysine 4 on histone H3 (H3K4me3) and active enhancers marked by H3K4me1 and H3K27Ac as well as a similar degree of overlap with Max binding sites (Figure 4C and Figure S4C), suggesting that Myc and NMyc play similar biological roles in ESCs. Approximately 50% of newly identified Myc binding sites by Bio-Myc ChIP-Seq overlap with NMyc targets and the newly identified Myc bound regions show enrichment in motif sequence corresponding to the perfect Myc E-Box with a definitely significant p-value (1E-142) (Figure 4D). Furthermore, gene ontology analysis showed that the newly identified genes are involved in cell cycle progression, cell metabolism, and molecular functions involved in the metabolism of nucleic acid, corresponding to the gene categories usually associated with Myc proteins (Figure 4E, F).

### The newly identified genes by Bio-Myc ChIP-Seq are actually bound by Myc in ES Cells

**Figure 5:**
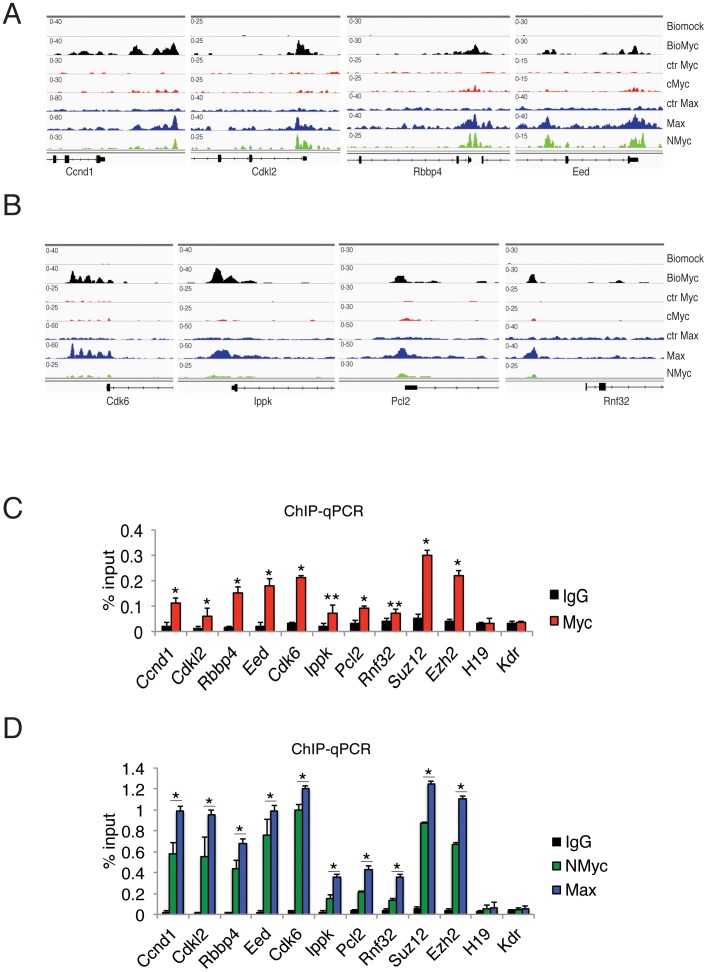
The newly identified genes by Biotag-Myc ChIP-Seq are bound by endogenous Myc in ES cells. (A–B) Representative genomic occupancy profiles of genes identified by Bio-Myc ChIP-Seq but not by endogenous Myc ChIP-Seq. The genes were divided in two groups. The first group is composed of the genes that are also bound by NMyc (A) while the second group is composed of the genes that are bound only by Bio-Myc (B). (C) RT-qPCR analysis of endogenous Myc ChIP samples at the promoters of the indicated genes. The Suz12 and Ezh2 genes were used as positive controls, and the H19 and Kdr genes were used as negative controls. The results are shown as the percentage (1/100) of the input. (D) RT-qPCR analysis of endogenous NMyc and Max ChIP reveals their binding to the promoters of the second group of genes. The Suz12 and Ezh2 genes were used as positive controls, the H19 and Kdr genes were used as negative controls. The results are shown as the percentage (1/100) of the input.

To further investigate the genes identified by Bio-Myc ChIP-Seq analysis that were not identified using the anti-Myc antibody, we analyzed the effective role of Myc in the regulation of these genes. We first compared the binding profiles of Bio-Myc, Myc, Max, and NMyc on two groups of genes. The first group is composed of the genes that are also bound by NMyc (Figure 5A), while the second group is composed of the genes that are bound only by Bio-Myc, but not by NMyc (Figure 5B). The genomic occupancy profiles revealed a very similar binding pattern of Bio-Myc and Max to the promoters of the second group of genes, and they showed an analogous binding pattern of NMyc for the first group genes. Interestingly, in the Myc ChIP-Seq of the first group of genes (Figure 5A) as well as in the NMyc ChIP-Seq of the second group genes (Figure 5B), several sporadic reads corresponding to the peak of Bio-Myc on gene promoters are evident. Although these signals are below the general background level, these reads may indicate the detection of Myc binding sites by the anti-Myc antibody. However, the sensitivity of the ChIP-Seq technique does allow the identification of these low signals as true peaks. To verify Myc binding at these genes, we performed ChIP followed by RT-qPCR analysis to reveal the binding of endogenous Myc. We noted a significant enrichment of Myc by ChIP at all regions that were identified by Bio-Myc ChIP-Seq in comparison with IgG and the negative controls (H19 and Kdr gene) (Figure 5C). In addition, ChIP analysis also revealed NMyc and Max binding to the chromatin of these genes (Figure 5D).

**Figure 6:**
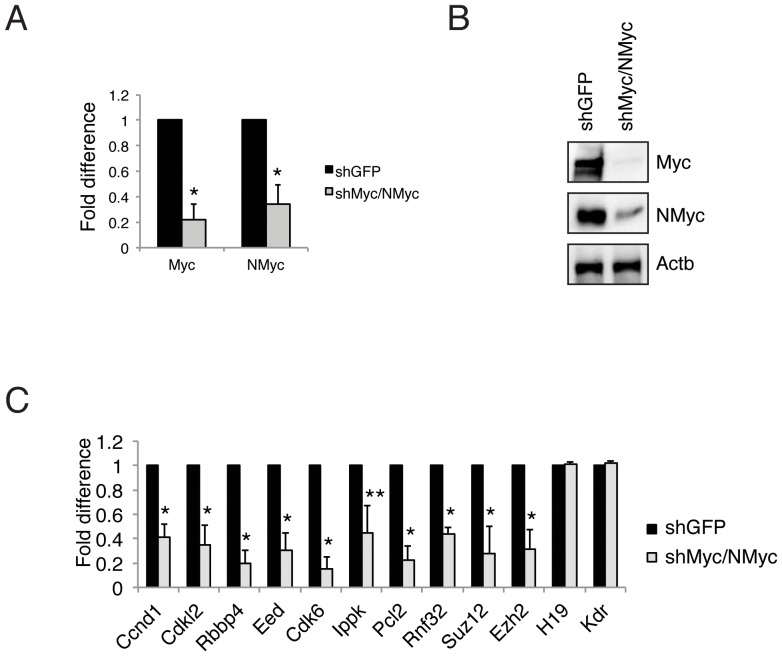
The genes identified by Biotag-Myc are regulated by Myc in ES cells. (A) RT-PCR analysis of Myc and NMyc expression in double knockdown ES cells. The results are shown as a fold difference. (B) Western blot analysis of Myc protein level in double-knockdown ES cells. β-actin was used as a loading control. (C) RT-PCR analysis of the indicated transcripts upon a double knockdown (dKD) of Myc proteins. The Suz12 and Ezh2 genes were used as positive controls, and the H19 and Kdr genes were used as negative controls. The results are shown as the fold difference.

We next verified whether these newly identified genes bound by Myc are actually regulated by Myc. To this end, we silenced the Myc protein in ES cells (Figure 6A and B). In agreement with previous data [8], knockdown of Myc resulted in the downregulation of the expression of Suz12 and Ezh2, which were used as controls (Figure 6C). Together with Suz12 and Ezh2, all the new genes identified by Bio-Myc were also down regulated, including those that were not identified by NMyc (Figure 6C), demonstrating that Myc positively regulate these genes. Because these genes are involved in the cell cycle, cell metabolism, and in the maintenance of the chromatin signature in ES cells, the knowledge of their regulation by Myc could allow a better understanding of the biological role of Myc in ES cells.

Using biotin-tagged Myc, we identified a more comprehensive genome-wide analysis of the Myc binding sites in ESCs than those obtained with Myc specific-antibodies. This approach could be employed in general for the identification of the binding profile of any chromatin-associated protein. The use of the Biotag also demonstrated the advantage of a better comparison between the different factors and the possibility to analyze the binding activities of mutants or alternative splice variants, which could not be distinguished by specific antibodies.

**Figure 7:**
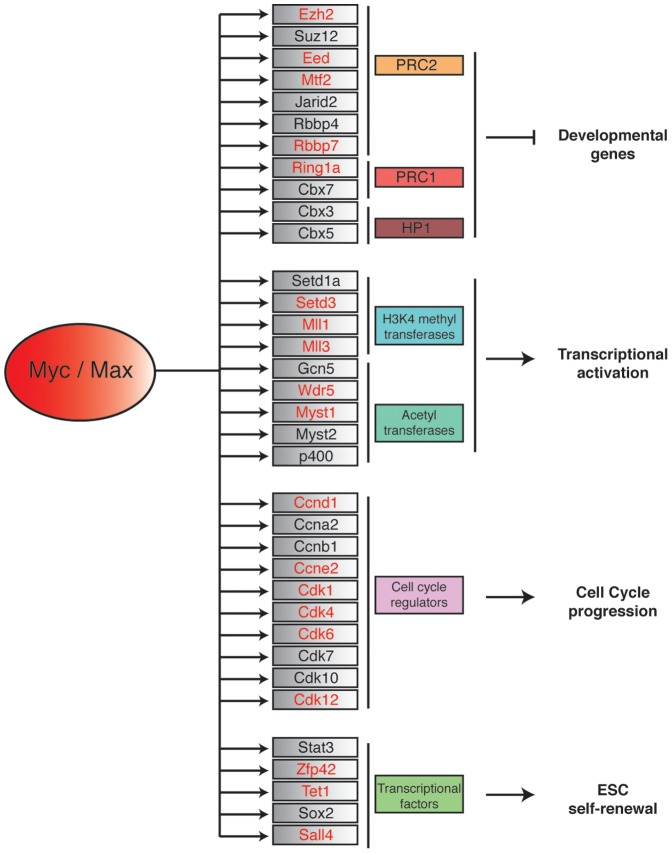
Myc/Max transcriptional targets in ESCs. Schematic representation of Myc/Max bound genes in ES cells (the genes identified in this work are indicated in red) and their molecular and biological functions.

To bind to the E-Box element and regulate its target genes, Myc must form a dimer with its partner Max. Importantly, to maintain ESC pluripotency, Myc requires the expression of Max [26]. From the data obtained, we generated a Myc/Max-dependent regulatory network that integrates previously identified Myc-genes with the genes identified in this study (Figure 7). The model includes a subset of representative Myc/Max co-bound targets involved in transcriptional activation, cell cycle progression, ESC self-renewal, and developmental processes. In line with the view of Myc as a general transcriptional activator, in addition to the previously identified Myc-target genes Gcn5, Myst2, and P400, we found other histone modifying factors that positively regulate transcription, such as Setd3, Mll1, Mll3, and Wdr5. Interestingly, our study also identified other categories of ES cell regulators.

## Conclusions

Previous genome-wide analysis of the Myc binding sites in ESCs suggested that the Myc-centered regulatory network is largely independent from the networks controlled by the core pluripotent factors and from Polycomb because each of these networks (or modules) regulates largely independent sets of target genes with distinct roles in maintaining ES cell self-renewal, undifferentiation, and proliferation [10]. Our genome-wide study also identified as Myc-targets the ESC-specific genes Stat3, Sox2, and Zfp42 (Rex1), which are factors involved in maintaining ESC pluripotency. This analysis also identified additional components of the Polycomb PRC1 and PRC2 complexes, which maintain the transcription of developmental genes in ES cells. Thus, our study suggests that the Myc-centered regulatory network, the core, and the Polycomb modules are more interconnected than previously thought, providing an explanation for the observation that Myc plays an essential role in maintaining ESC self-renewal and the undifferentiated state [6] [8].

## Supporting Information

Figure S1 **Characterization and Functional Validation of Tagged c-Myc Stable Clones.** (A) Western blot analysis of the exogenous tagged c-myc expression in the indicated stable clones. The level of expression was determined by using the antibody 9e10. Beta-actin was used as a loading control. (B–D) Western blot analysis of affinity tags expression in the indicated stable clones. Beta-actin was used as a loading control. (E) Immunoprecipitation analysis of endogenous Max protein in nuclear extracts obtained from the indicated clones demonstrated that Max is able to coimmunoprecipitate with exogenous Myc in each clone analysed. Purified rabbit IgG were used as negative control. (F) Immunoprecipitation analysis of exogenous Myc using the corresponding affinity beads showed its interaction with endogenous Max in each clone analysed.
(TIF)

Figure S2 **Analysis of Biotag and Flag-HA ChIP Assays.** (A) RT-PCR analysis of Bio-Myc_5B ChIP performed under three different concentrations of SDS (0.15%, 0.5% and 1%) in lysis buffer. The Suz12 and Ezh2 genes were used as positive controls, the H19 and Kdr genes were used as negative controls. The results are shown as a fold difference. (B) RT-PCR analysis of Flag ChIP performed in two different Flag-HA-Myc stable clones. Two different concentrations of SDS (0.15%, 0%) in lysis buffer, and two different types of elution for each condition were used as indicated. The Suz12 and Ezh2 genes were used as positive controls, the H19 and Kdr genes were used as negative controls. The results are shown as a fold difference. (C) RT-PCR analysis of TAP ChIP performed in two different Flag-HA-Myc stable clones. Flag-HA-Myc was first immunoprecipitated with anti-Flag M2 beads and then it was reimmunoprecipitated using anti-HA affinity gel. The immunoprecipitation was performed in lysis buffer containing 0% of SDS. Two different types of elution were used as indicated. The Suz12 and Ezh2 genes were used as positive controls, the H19 and Kdr genes were used as negative controls. The results are shown as a fold difference.
(TIF)

Figure S3 **Analysis of V5 ChIP Assay.** (A) RT-PCR analysis of V5-Myc_3A ChIP performed under three different concentrations of SDS (0.15%, 0.5% and 1%) in lysis buffer, and two different types of elution for each condition. The Suz12 and Ezh2 genes were used as positive controls, the H19 and Kdr genes were used as negative controls. The results are shown as a fold difference. (B) RT-PCR analysis of V5 ChIP performed in two different V5-Myc stable clones under the indicated concentration of SDS in lysis buffer. V5-Myc was immunoprecipitated either with V5 magnetic beads or with anti-V5 antibody and two different types of elution for each condition were performed. The Suz12 and Ezh2 genes were used as positive controls, the H19 and Kdr genes were used as negative controls. The results are shown as a fold difference. (C) RT-PCR analysis of V5 ChIP performed in two different V5-Myc stable clones under the indicated concentration of SDS in lysis buffer. V5-Myc was immunoprecipitated with V5 magnetic beads and two different types of elution for each condition were performed. The Suz12 and Ezh2 genes were used as positive controls, the H19 and Kdr genes were used as negative controls. The results are shown as a fold difference.
(TIF)

Figure S4 **Comparison of Different Types of Affinity Tags for Use in Chromatin Immunoprecipitation.** (A) RT-qPCR analysis of ChIP assays of the indicated endogenous and tagged proteins under the conditions indicated. The Suz12 and Ezh2 genes were used as positive controls, the H19 and Kdr genes were used as negative controls. The results are shown as percentage (1/100) of input. (B) Backround average level of the indicated ChIP assays shown as percentage (1/100) of input. Background was calculated as the average of the values obtained from mock or IgG ChIP on two positive (Suz12, Ezh2) and two negative (H19, Kdr) regions plus the values of the indicated ChIP on the two negative regions (H19, Kdr). (C) Overlap between Myc binding regions and promoters and enhancers with the indicated features.
(TIF)

Figure S5 **Analysis of the Myc, Core and Polycomb modules target genes in ESC.** (A) Venn diagram showing the exact number of promoters bound in the indicated ChIP-Seq analyses. Since Oct3/4 and Polycomb could bind not exactly on TSS, the analysis was performed keeping the genes bound in a larger region near TSS (between -3kb and +2kb). (B) Clustering analysis of the global Pearson correlation of the indicated ChIP-Seq showing the independence of the three modules in ESC.
(TIF)

Table S1 **ChiP primers.**
(DOCX)

Table S2 **RT-PCR primers.**
(DOCX)
