# Supplementary figures and images for: Myc and Max Genome-Wide Binding Sites Analysis Links the Myc Regulatory Network with the Polycomb and the Core Pluripotency Networks in Mouse Embryonic Stem Cells
Source: PLoS One. 2014 Feb 21;9(2):e88933. doi: 10.1371/journal.pone.0088933 (PMC3931652; doi:10.1371/journal.pone.0088933)

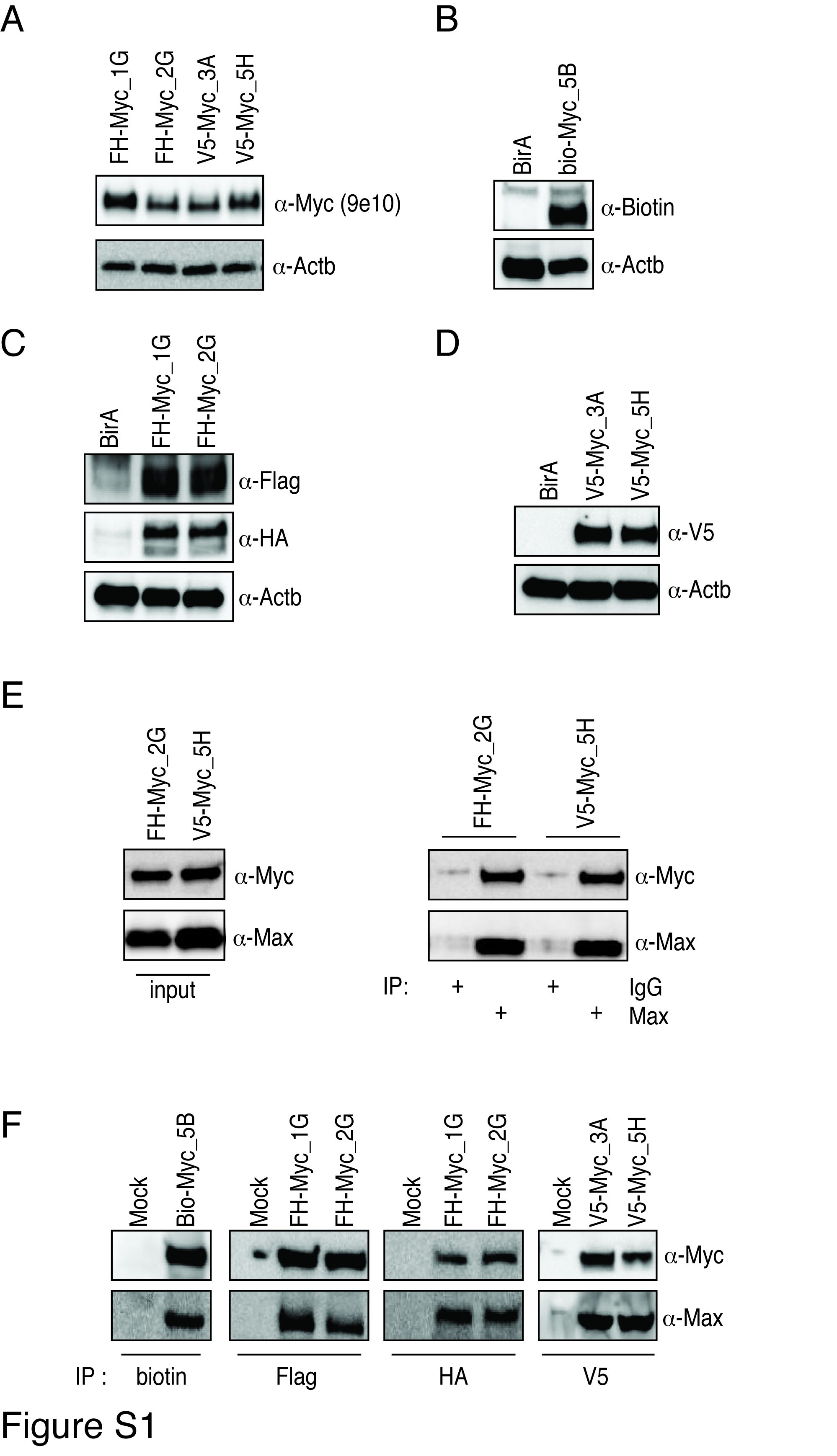

Supplement: Figure S1 — Characterization and Functional Validation of Tagged c-Myc Stable Clones. (A) Western blot analysis of the exogenous tagged c-myc expression in the indicated stable clones. The level of expression was determined by using the antibody 9e10. Beta-actin was used as a loading control. (B–D) Western blot analysis of affinity tags expression in the indicated stable clones. Beta-actin was used as a loading control. (E) Immunoprecipitation analysis of endogenous Max protein in nuclear extracts obtained from the indicated clones demonstrated that Max is able to coimmunoprecipitate with exogenous Myc in each clone analysed. Purified rabbit IgG were used as negative control. (F) Immunoprecipitation analysis of exogenous Myc using the corresponding affinity beads showed its interaction with endogenous Max in each clone analysed. (TIF) [file pone.0088933.s001.tif]

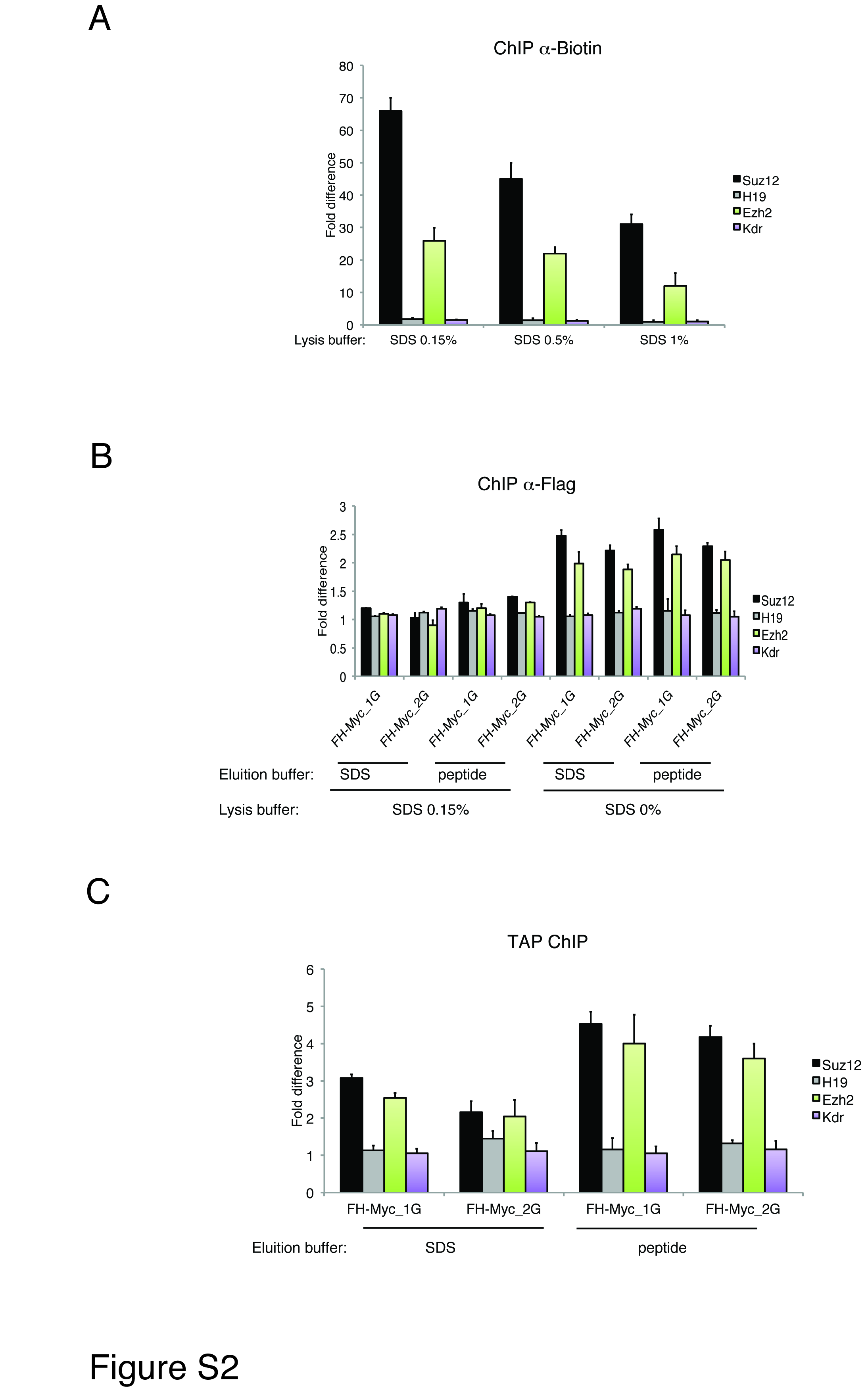

Supplement: Figure S2 — Analysis of Biotag and Flag-HA ChIP Assays. (A) RT-PCR analysis of Bio-Myc_5B ChIP performed under three different concentrations of SDS (0.15%, 0.5% and 1%) in lysis buffer. The Suz12 and Ezh2 genes were used as positive controls, the H19 and Kdr genes were used as negative controls. The results are shown as a fold difference. (B) RT-PCR analysis of Flag ChIP performed in two different Flag-HA-Myc stable clones. Two different concentrations of SDS (0.15%, 0%) in lysis buffer, and two different types of elution for each condition were used as indicated. The Suz12 and Ezh2 genes were used as positive controls, the H19 and Kdr genes were used as negative controls. The results are shown as a fold difference. (C) RT-PCR analysis of TAP ChIP performed in two different Flag-HA-Myc stable clones. Flag-HA-Myc was first immunoprecipitated with anti-Flag M2 beads and then it was reimmunoprecipitated using anti-HA affinity gel. The immunoprecipitation was performed in lysis buffer containing 0% of SDS. Two different types of elution were used as indicated. The Suz12 and Ezh2 genes were used as positive controls, the H19 and Kdr genes were used as negative controls. The results are shown as a fold difference. (TIF) [file pone.0088933.s002.tif]

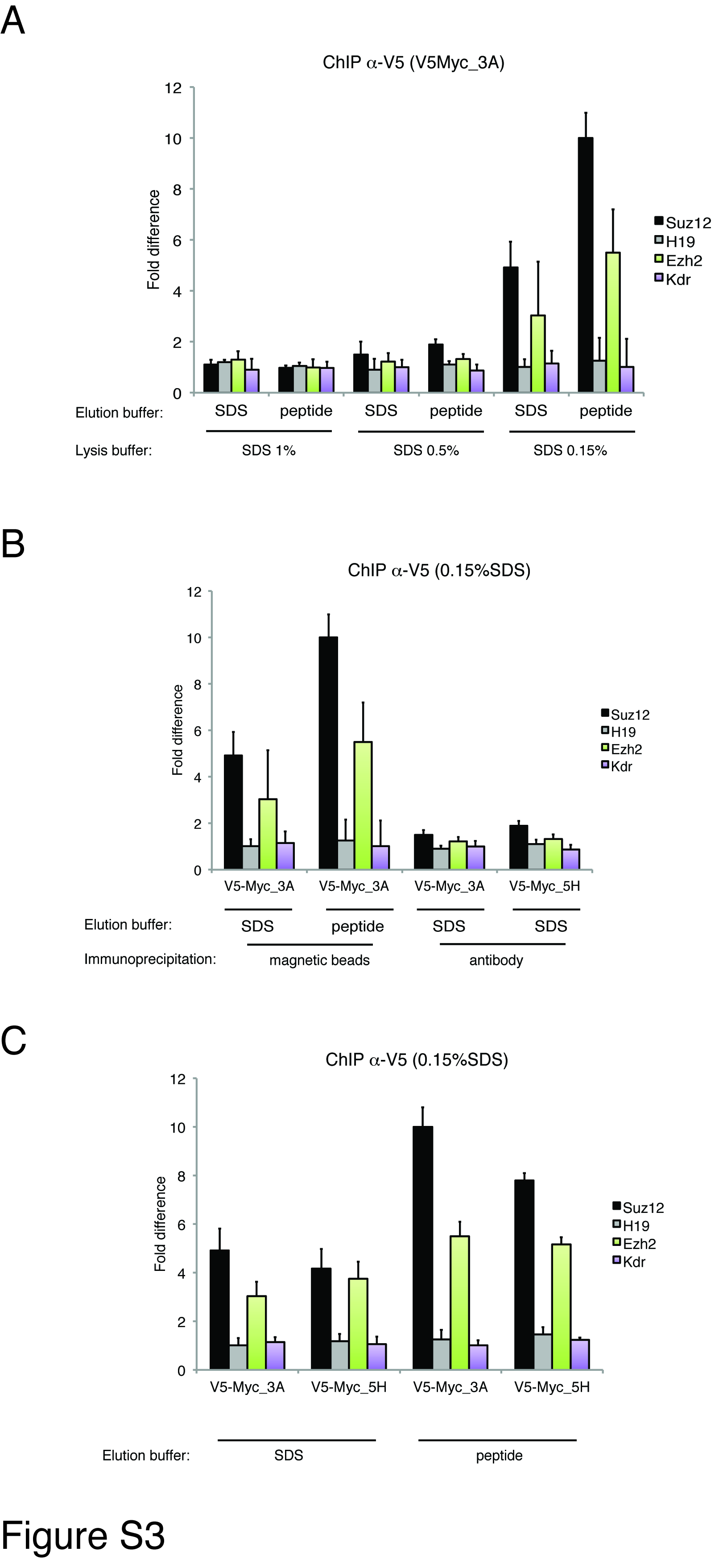

Supplement: Figure S3 — Analysis of V5 ChIP Assay. (A) RT-PCR analysis of V5-Myc_3A ChIP performed under three different concentrations of SDS (0.15%, 0.5% and 1%) in lysis buffer, and two different types of elution for each condition. The Suz12 and Ezh2 genes were used as positive controls, the H19 and Kdr genes were used as negative controls. The results are shown as a fold difference. (B) RT-PCR analysis of V5 ChIP performed in two different V5-Myc stable clones under the indicated concentration of SDS in lysis buffer. V5-Myc was immunoprecipitated either with V5 magnetic beads or with anti-V5 antibody and two different types of elution for each condition were performed. The Suz12 and Ezh2 genes were used as positive controls, the H19 and Kdr genes were used as negative controls. The results are shown as a fold difference. (C) RT-PCR analysis of V5 ChIP performed in two different V5-Myc stable clones under the indicated concentration of SDS in lysis buffer. V5-Myc was immunoprecipitated with V5 magnetic beads and two different types of elution for each condition were performed. The Suz12 and Ezh2 genes were used as positive controls, the H19 and Kdr genes were used as negative controls. The results are shown as a fold difference. (TIF) [file pone.0088933.s003.tif]

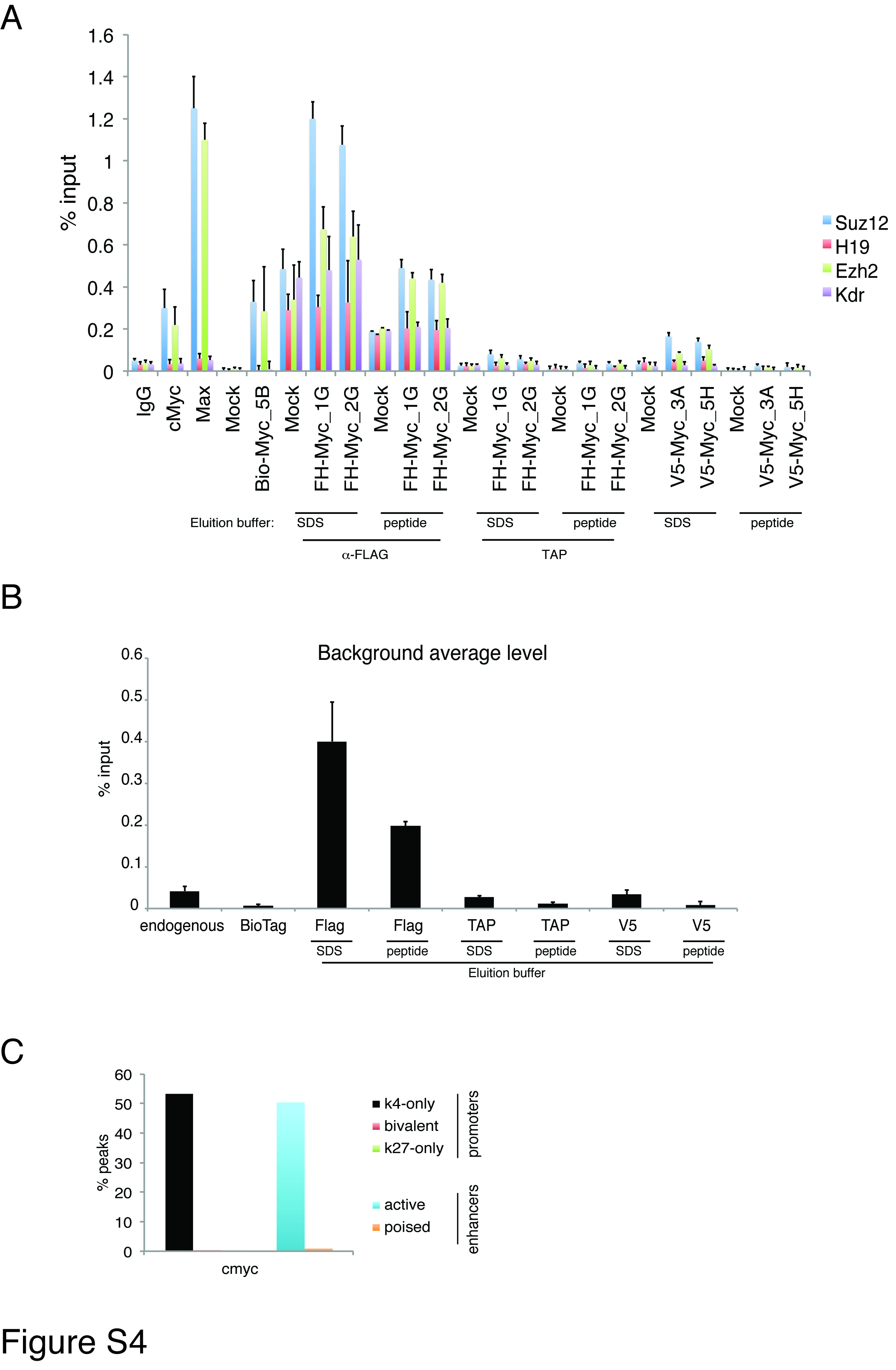

Supplement: Figure S4 — Comparison of Different Types of Affinity Tags for Use in Chromatin Immunoprecipitation. (A) RT-qPCR analysis of ChIP assays of the indicated endogenous and tagged proteins under the conditions indicated. The Suz12 and Ezh2 genes were used as positive controls, the H19 and Kdr genes were used as negative controls. The results are shown as percentage (1/100) of input. (B) Backround average level of the indicated ChIP assays shown as percentage (1/100) of input. Background was calculated as the average of the values obtained from mock or IgG ChIP on two positive (Suz12, Ezh2) and two negative (H19, Kdr) regions plus the values of the indicated ChIP on the two negative regions (H19, Kdr). (C) Overlap between Myc binding regions and promoters and enhancers with the indicated features. (TIF) [file pone.0088933.s004.tif]

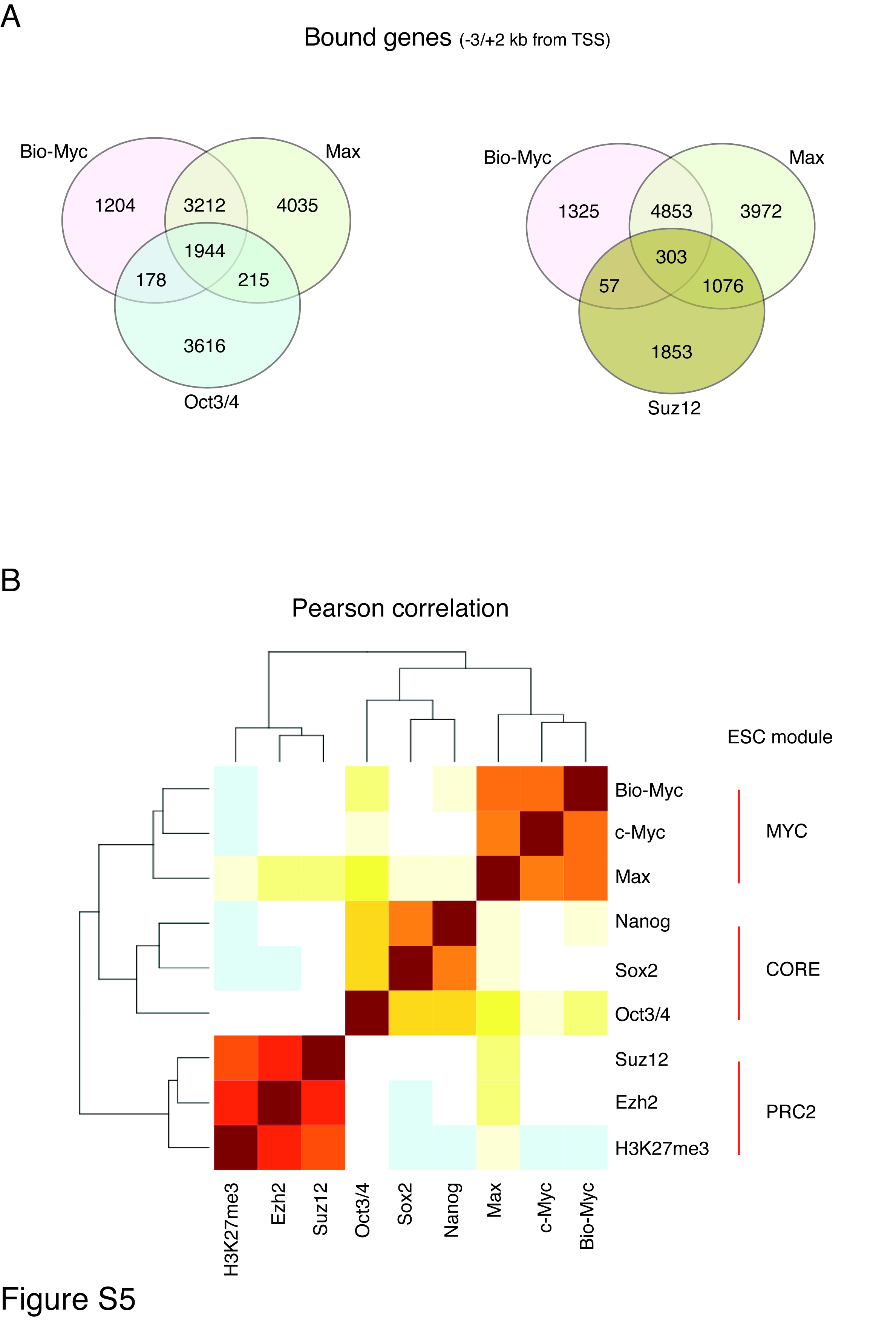

Supplement: Figure S5 — Analysis of the Myc, Core and Polycomb modules target genes in ESC. (A) Venn diagram showing the exact number of promoters bound in the indicated ChIP-Seq analyses. Since Oct3/4 and Polycomb could bind not exactly on TSS, the analysis was performed keeping the genes bound in a larger region near TSS (between -3kb and +2kb). (B) Clustering analysis of the global Pearson correlation of the indicated ChIP-Seq showing the independence of the three modules in ESC. (TIF) [file pone.0088933.s005.tif]
