# Supplementary material for: Myc and Max Genome-Wide Binding Sites Analysis Links the Myc Regulatory Network with the Polycomb and the Core Pluripotency Networks in Mouse Embryonic Stem Cells
Source: PLoS One. 2014 Feb 21;9(2):e88933. doi: 10.1371/journal.pone.0088933 (PMC3931652; doi:10.1371/journal.pone.0088933)
Supplement: Table S2 — RT-PCR primers. (DOCX) [file pone.0088933.s007.docx]

**Table S2. Primers used for RT-PCR analysis**
